# Development of a Novel Loop-Mediated Isothermal Amplification Method to Detect Guiana Extended-Spectrum (GES) β-Lactamase Genes in *Pseudomonas aeruginosa*

**DOI:** 10.3389/fmicb.2019.00025

**Published:** 2019-02-04

**Authors:** Chika Takano, Mitsuko Seki, Dong Wook Kim, Humphrey Gardner, Robert E. McLaughlin, Paul E. Kilgore, Kazunari Kumasaka, Satoshi Hayakawa

**Affiliations:** ^1^Division of Microbiology, Department of Pathology and Microbiology, Nihon University School of Medicine, Tokyo, Japan; ^2^Division of Pediatric Dentistry, Meikai University School of Dentistry, Sakado, Japan; ^3^Department of Pharmacy, College of Pharmacy, Hanyang University, Ansan, South Korea; ^4^Institute of Pharmacological Research, Hanyang University, Ansan, South Korea; ^5^Evelo Biosciences, Cambridge, MA, United States; ^6^Institute for Life Science Entrepreneurship, Union, NJ, United States; ^7^Department of Pharmacy Practice, Eugene Applebaum College of Pharmacy and Health Sciences, Wayne State University, Detroit, MI, United States; ^8^Department of Laboratory Medicine, Ageo Central General Hospital, Ageo, Japan

**Keywords:** *bla*_GES_, β-lactamase, point mutation, carbapenemase, loop-mediated isothermal amplification, *Pseudomonas aeruginosa*

## Abstract

Infections caused by multidrug-resistant *Pseudomonas aeruginosa* in hospitalized patients are often fatal, and nosocomial infections caused by Guiana extended-spectrum (GES) β-lactamase-producing strains are of growing concern. Several genotypes of the GES β-lactamase gene (*bla*_GES_) include a single missense mutation, a change from G to A at nucleotide position 493 (G493A) that changes glycine to serine; the mutant enzyme exhibits carbapenemase activity. Rapid and reliable identification of drug-resistance is important in clinical settings; however, culture methods remain the gold standard. Conventional and real-time PCR cannot identify carbapenemase-producing genotypes, and direct DNA sequencing is essential. We established a novel loop-mediated isothermal amplification (LAMP) method to detect various genotypes of *bla*_GES_ and another LAMP method to discriminate carbapenemase genotypes of *bla*_GES_. We evaluated the two assays using clinical *P. aeruginosa* strains. Two primer sets targeting *bla*_GES_ (GES-LAMP) and the point mutation (Carba-GES-LAMP) were designed and evaluated for specificity and sensitivity. The detection limit of the GES-LAMP method was assessed using purified DNA and DNA-spiked clinical samples (urine, sputum, and blood). To determine the clinical usefulness of the methods, we used different (genotypically and phenotypically) *P. aeruginosa* clinical isolates, collected from diverse geographical locations between 2003 and 2012. The novel LAMP assay targeting *bla*_GES_ was highly specific. The detection limit was 10 DNA copies per reaction; the assay was 10-fold more sensitive than conventional PCR. The LAMP assay detected *bla*_GES_ with high sensitivity in all DNA-spiked samples; PCR did not detect *bla*_GES_ in blood samples. The GES-LAMP method correctly detected the 5 isolates containing *bla*_GES_ among the 14 isolates tested. Using these isolates, we confirmed that our Carba-GES-LAMP method of detecting point mutations correctly identified the two *bla*_GES_ positive organisms with carbapenemase activity. To the best of our knowledge, this is the first report of the GES β-lactamase gene detection assay using the LAMP method. Our new assays effectively detect *bla*_GES_ and critical unique mutations.

## Introduction

Multidrug-resistant bacteria have spread worldwide and become a major clinical concern and public health issue as well. Invasive infection by *Pseudomonas aeruginosa* is often fatal in the absence of appropriate antibiotic treatment (Miyoshi-Akiyama et al., 2017). As β-lactamase genes are plasmid-borne, a failure to treat drug-resistant bacteria may trigger nosocomial infections. Of the various β-lactamase genes, Guiana extended-spectrum (GES) β-lactamase has become of increasing concern (Poirel et al., 2012). GES was first described in 2000 in French Guiana (Poirel et al., 2000) and is an Amber class A extended-spectrum β-lactamase. However, certain variants (GES-4, -5, -6, -14, -15, -16, -18, -20, and -24) feature a single missense mutation at nucleotide position 493 (G493A) that changes glycine 165 to serine (Gly165Ser), which was previously reported as Gly170Ser (Bebrone et al., 2013). The resulting mutant enzyme exhibits carbapenemase activity (Bonnin et al., 2017). In Japan, GES-4 and GES-5 carbapenemase-producing *P. aeruginosa* have caused severe hospital-acquired infections (Kanayama et al., 2016; Yamasaki et al., 2017). Reports of GES-type enzymes remain rare but are steadily increasing (Naas et al., 2016).

Rapid and reliable identification of drug-resistance is essential to ensure that antibiotic use is appropriate. Conventional culture remains the gold standard for assessing antibiotic resistance despite being time-consuming, requiring sophisticated laboratory equipment and quality-control, and yielding ambiguous outcomes. Furthermore, the number of inoculated bacteria affect the drug minimum inhibitory concentration (MIC) (Bratu et al., 2005). Conventional PCR-based assays can detect β-lactamase genes but require well-equipped laboratories. Moreover, neither conventional PCR nor real-time PCR can identify carbapenemase-producing genotypes, and direct DNA sequencing is essential to do so (de Oliveira et al., 2017).

Loop-mediated isothermal amplification (LAMP) methods are becoming increasingly popular due to their relative simplicity and accuracy. The unique priming mechanism allows rapid and specific DNA amplification (Notomi et al., 2000), with no requirement for expensive equipment or a sophisticated laboratory. LAMP is a convenient and inexpensive alternative to PCR in terms of point-of-care testing (POCT). Here, we established two LAMP methods to detect the GES β-lactamase gene (*bla*_GES_, GES-LAMP) and carbapenemase genotypes (Carba-GES-LAMP) and applied the two assays to evaluate characterized (both genotypically and phenotypically) clinical *P. aeruginosa* strains from diverse geographical locations collected between 2003 and 2012 (Kos et al., 2015).

## Materials and Methods

### Bacterial Strains

A total of 22 bacterial strains including 8 standard strains (Table 1) and 14 clinical *P. aeruginosa* strains (Table 2) were used to evaluate the LAMP methods. The eight standard strains included six kinds of genotypes of β-lactamase producers (KPC, NDM, VIM, IMP, OXA, and GES): two *Klebsiella pneumoniae*, one *Escherichia coli*, four *P. aeruginosa*, and one *Acinetobacter bereziniae* provided by AstraZeneca (Waltham, MA, United States). Genomic DNA was extracted using the Maxwell 16-cell DNA purification kit (Promega, Madison, WI, United States). DNA concentrations were measured using the NanoDrop 1000 (Thermo Fisher Scientific Inc., Waltham, MA, United States). Genome copy numbers were calculated based on genome sizes of 6.5 Mbp for *P. aeruginosa* (PB369; GenBank accession number, CP025049.1), 5.4 Mbp for *K. pneumoniae* (Kp52.145; GenBank accession number, FO834906.1), 5.2 Mbp for *E. coli* (CFT073; GenBank accession number, AE014075.1), and 4.5 Mbp for *A. bereziniae* (XH901; GenBank accession number, NZ_CP018259.1). Each DNA sample was normalized to the same concentration and used to evaluate assay specificity. To validate *bla*_GES_ detection limits, we used genomic DNA from the *P. aeruginosa* strain ARC3917. Serial 10-fold-diluted DNA samples (10^5^, 10^4^, 10^3^, 10^2^, 10, and 1 genome copies) were amplified by LAMP and the results were compared with those from PCR assays. To confirm reproducibility, triplicate tests were performed over a 3-day period.

**Table 1:** Reactivities and specificities of PCR and LAMP assays detecting *bla*_GES_.

Strain ID	Species	Genotype	PCR^a^	GES-LAMP^b^
ARC2780	*Acinetobacter bereziniae*	IMP-1	(-)^c^	(-)
ARC2945	*Klebsiella pneumoniae*	KPC-2	(-)	(-)
ARC3471	*Pseudomonas aeruginosa*	VIM-2	(-)	(-)
ARC3475	*Pseudomonas aeruginosa*	OXA-48	(-)	(-)
ARC3600	*Escherichia coli*	NDM-1	(-)	(-)
ARC3802	*Klebsiella pneumoniae*	NDM-1	(-)	(-)
ARC3917	*Pseudomonas aeruginosa*	GES-1	(+)	(+)
ARC3936	*Pseudomonas aeruginosa*	VIM-7	(-)	(-)


*^a^PCR results obtained via electrophoretic analysis; ^b^LAMP results determined via Loopamp real-time turbidimetry and the naked eye. ^c^ (-), negative; (+), positive.*

**Table 2:** Clinical *Pseudomonas aeruginosa* isolates evaluated.

Strain no.	Origin of isolate		Genotype	Meropenem		Assays		
	Country	Anatomical site		MIC (mg/L)		PCR	GES-LAMP	Carba-GES-LAMP
**GES β-lactamase-producing strains**								
AZPAE14831	Argentina	RTI^a^	GES-1	0.5	(S)^d^	(+)	(+)	(-)
AZPAE14948	Argentina	IAI^b^	GES-5	>32	(R)	(+)	(+)	(+)
AZPAE13856	India	Unknown	GES-7	0.5	(S)	(+)	(+)	(-)
AZPAE13848	India	Unknown	GES-9	0.25	(S)	(+)	(+)	(-)
AZPAE13880	Mexico	Unknown	OXA-2, GES-19, GES-20-like	>32	(R)	(+)	(+)	(+)
**Other β-lactamase-producing strains**								
AZPAE13872	Mexico	Unknown	IMP-15	>32	(R)	(-)	(-)	(-)
AZPAE13879	Argentina	Unknown	VIM-11, OXA-17	16	(R)	(-)	(-)	(-)
AZPAE14688	Mexico	Unknown	IMP-18	>32	(R)	(-)	(-)	(-)
AZPAE14719	Colombia	RTI	KPC-2	>32	(R)	(-)	(-)	(-)
AZPAE14720	Colombia	UTI^c^	OXA-2, KPC-2	>32	(R)	(-)	(-)	(-)
AZPAE14822	Brazil	IAI	OXA-56	8	(R)	(-)	(-)	(-)
AZPAE14862	India	UTI	IMP-13	2	(S)	(-)	(-)	(-)
AZPAE14900	India	IAI	OXA-10, VEB-like, VIM-5	16	(R)	(-)	(-)	(-)
AZPAE15029	France	RTI	VIM-2, OXA-4	>32	(R)	(-)	(-)	(-)


*^a^Respiratory tract infections; ^b^Urinary tract infections, ^c^Intra-abdominal infections. ^d^ (S), susceptible; (R), resistant.*

### Clinical *P. aeruginosa* Strains

Fourteen clinical *P. aeruginosa* strains including five *bla*_GES_ segments (GES-1, -5, -7, -9, and -19/20-like) were randomly selected from 388 strains with previously reported genotypes and phenotypes (Kos et al., 2015; Table 2) isolated from diverse geographical locations (Colombia, India, Spain, France, Greece, Germany, Argentina, Croatia, China, Brazil, Mexico, and the Philippines) between 2003 and 2012. Genomic DNA was extracted using the Maxwell 16-cell DNA purification kit (Promega). Whole-genome sequences were analyzed using the HiSeq 2000 or MiSeq platforms (Illumina, San Diego, CA, United States). Susceptibility to meropenem was explored using frozen Trek-Sensititre custom plates (Thermo Fisher Scientific Inc.) following the guidelines of the Clinical and Laboratory Standards Institute (2012). The results of meropenem MIC are listed in Table 2. We used genomic DNA from *P. aeruginosa* strain AZPAE4948 to evaluate the LAMP assay.

### LAMP Primer Design

We targeted the *bla*_GES_ gene (GES-1; GenBank accession number, AF355189.1; Supplementary Figure S1) using primers designed by Primer Explore V5 software (FUJITSU, 2016). The LAMP primers included two outer primers (F3 and B3), a forward inner primer (FIP), a backward inner primer (BIP), and loop primers (LF and LB) (Table 3). In addition, we developed primers targeting the G493A mutation of *bla*_GES_ (GES-5; GenBank accession number, EF190326.1; Supplementary Figure S1) using an amplification-refractory mutation system (ARMS) (Newton et al., 1989; Ikeda et al., 2007). The BIP featured the addition of a single nucleotide mutation to the 5′-end, followed by the addition of two mutations in the second (G to C) and fifth (A to T) positions from the 5′ end. Other primers were designed using Primer Explorer V5 software (Table 3).

**Table 3:** LAMP primer sets used for genotypic identification of *bla*_GES_ and the single missense mutation (G493A) that changes glycine 165 to a serine, endowing the enzyme with carbapenemase activity.

	Sequence 5′–3′
GES-LAMP primer	
GES_F3	ACC ATT GAG AGG TGG CTG AT
GES_B3	TGA CCG ACA GAG GCA ACT
GES_FIP	GTT GGC GCA GGT ACC AGT TTT CCG ACA CTA CGA GCG GGT T
GES_BIP	GCC CAG GAG AGA GAT TAC GCT GAT TCG TCA CGT TCT ACG GC
GES_LF	TCT CCA ACA ACC CAA TCT TTA GG
GES_LB	GTG TAT ACA ACG GCC CCG A
Carba-GES-LAMP primer	
Carba-GES_F3	TGC AGC TTA GCG ACA ATG G
Carba-GES_B3	CCG CCA TAG AGG ACT TTA GC
Carba-GES_FIP	AGC CGA CTC ACA GAG TCG CCA GAG AAA TTG GCG GAC CTG
Carba-GES_BIP	**A**^a^**C**^b^C G**T**^c^C AAC ACA CCT GGC GAC ACA GTA CGT GCC ATA GCA A
Carba-GES_LF	CGA AAA TAC TGC GTC ATT GCA G
Carba-GES_LB	CCT CAG AGA TAC AAC TAC GCC TA


*^a^A single missense mutation (G493A) detected by ARMS; ^b^original sequence G; ^c^original sequence A.*

### LAMP

The LAMP reaction mixture (25 μL) contained 1.6 μM FIP and BIP each, 0.2 μM F3 and B3 each, 0.4 μM LF, 8 U *Bst* DNA polymerase (large fragment) (New England Biolabs, Ipswich, MA, United States), 1.4 mM all four deoxynucleoside triphosphates, 0.8 M betaine (Sigma, St. Louis, MO, United States), 20 mM Tris–HCl (pH 8.8), 10 mM KCl, 10 mM (NH_4_)_2_SO_4_, 8 mM MgSO_4_, 0.1% (v/v) Tween 20, and template DNA (2 μL). Each mixture was incubated at 63°C for 60 min and then heated at 80°C for 2 min to terminate the reaction. For the Carba-GES-LAMP assay, the incubation time was 50 min. We monitored reaction tube turbidity in real time using a Loopamp turbidimeter (EXIA; Eiken Chemical Co., Tokyo, Japan) to read the optical density at 650 nm (OD_650_) at 6-s intervals. We recorded the time required to exceed a turbidity level of 0.1, in accordance with the manufacturer’s protocol. Amplified products could be seen with the naked eye.

### Analysis of LAMP Products

Amplified LAMP products were sequenced at the Akita Prefectural University Biotechnology Center using the BigDye Terminator V3.1 cycle sequencing kit (Applied Biosystems, Foster City, CA, United States) on the 3130xL genetic analyzer (Applied Biosystems). The following F2 primers for GES-LAMP and Carba-GES-LAMP were used to sequence the target regions; GES-F2, 5′-TTC TAG CAT CGG GAC ACA TG-3′ and Carba-GES-F2, 5′-AGA GAA ATT GGC GGA CCT G-3′, respectively.

### PCR

*bla*_GES_ genes were amplified by PCR using previously described primers: GES-F, 5′-CTA TTA CTG GCA GGG ATC G-3′; GES-R, 5′-CCT CTC AAT GGT GTG GGT-3′ (Monteiro et al., 2012). PCR assays were performed using the Ex Taq enzyme (Takara Bio, Tokyo, Japan) in a 25 μL reaction mixture containing 0.2 mM each deoxyribonucleoside triphosphate, 10 mM Tris–HCl buffer (pH 8.3), 50 mM KCl, 2 mM MgCl_2_, 0.5 μM each primer, 1 U Ex Taq DNA polymerase, and template DNA (2 μL). The PCR program sequence was 94°C for 1 min, followed by 30 cycles of denaturation at 94°C for 30 s, annealing at 55°C for 30 s, and extension at 72°C for 60 s, and a final extension at 72°C for 15 min, followed by storage at 4°C. All reactions were performed in duplicate using the Veriti thermal cycler (Applied Biosystems). The resulting PCR products were subjected to agarose gel electrophoresis followed by staining with ethidium bromide. The expected size of the DNA fragment is 594 bp.

### DNA-Spiked Specimens

To analyze the effects of biological substances on the established LAMP amplification, we studied the sensitivity of the LAMP assay using DNA-spiked clinical samples. We collected urine and blood specimens from five healthy volunteers in Nihon University School of Medicine. The blood specimens were heparinised and stored at -80°C. Sputum specimens were obtained from seven patients of the Ageo Central General Hospital and frozen at -80°C to inactivate bacteria. After approval was granted by the Biosafety Committee of Nihon University, the specimens were handled using the risk group 2 protocol of the laboratory biosafety manual of the World Health Organization, Geneva, 2004 (World Health Organization, 2004).

Urine specimens were boiled at 95°C for 5 min and then centrifuged at 1,500 rpm. Blood and sputum samples were subjected to a Loopamp^TM^ PURE DNA extraction kit (Eiken Chemical Co.) according to the manufacturer’s instructions. Purified *bla*_GES_ DNA (from *P. aeruginosa* ARC3917) was spiked into the specimens and used to determine the detection limits of the GES-LAMP and PCR assays.

### Ethics Statement

We utilized urine and blood specimens from five healthy volunteers in Nihon University School of Medicine. The study protocol was reviewed and approved by the Institutional Review Board of Nihon University School of Medicine (IRB # 28-9-0). Written informed consent was obtained from five healthy volunteers. Using the IRB approved protocol, seven patient sputum specimens (Ageo Central General Hospital) were collected in accordance with the recommendations of the Japan Society of Clinical Examination Medicine that supports “the use of specimens that have completed clinical tests for work, education, research.” This guidance provides access to specimens when it is difficult to obtain consent, the sample is anonymized, and where the scientific/ethical review committee (i.e., IRB) has approved the study protocol. Written consent was waived because specimens were anonymized discarded samples from the hospital clinical laboratory. The ethical approval for collection of those specimens were obtained from the ethical committee at the Ageo Central General Hospital (Approval # 434) and the Institutional Review Board of Nihon University School of Medicine (Approval # 28-9-0).

## Results

### Analytical Reactivity and Specificity of the GES-LAMP Assay

**FIGURE 1:**
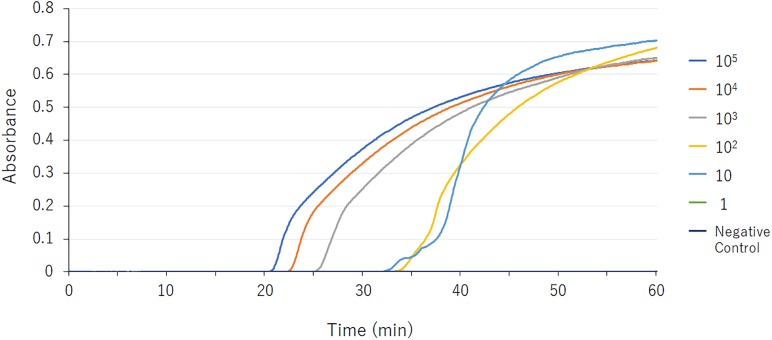
*bla*_GES_ LAMP assay data derived via real-time turbidimetry. Serial 10-fold-diluted samples (10^5^, 10^4^, 10^3^, 10^2^, 10, and 1 DNA copies) were assayed by LAMP. The detection limit was 10 DNA copies.

The LAMP assay successfully amplified the target sequence, as confirmed by visually evident turbidity in the reaction tube and by real-time turbidimetry (Figure 1). Of the various β-lactamase genes, the assay detected only *bla*_GES_ (Table 1). The product was subjected to direct sequencing. The sequences were compared with those of the targeted region (bases 337–425) of the GES-1 gene (from F1 to B1c, Supplementary Figure S1A). The sequences obtained were identical to those expected (Supplementary Figure S2A).

### Detection Limits

The detection limit of the GES-LAMP assay was 10 DNA copies per reaction, and that of the PCR assay was 100 copies. The LAMP assay was thus highly sensitive, 10-fold more so than PCR. LAMP products were measurable turbidimetrically in real-time and were evident with the naked eye. No false-positive reactions were observed.

### DNA-Spiked Specimens

Assay detection limits were determined using DNA-spiked blood, urine, and sputum. The GES-LAMP detection limits were 10 DNA copies per reaction, thus identical to that reported above (Table 4). The PCR detection limits were 100 DNA copies for the DNA-spiked urine and sputum specimens, again identical to that reported above. However, the detection limit was >10^5^ copies in DNA-spiked blood specimens (Table 4).

**Table 4:** Detection limits of the PCR and LAMP assays used to detect DNA from *P. aeruginosa* of genotype *bla*_GES_ in DNA-spiked specimens.

	Detection limit	
	PCR	GES-LAMP
Purified DNA	10^2^ copies^a^	10
**DNA spiked specimens**		
Urine^b^	10^2^	10
Sputum^c^	10^2^	10
Blood^c^	>10^5^	10


*^a^Amount of DNA per reaction; ^b^Supernatant data obtained after boiling and centrifugation; ^c^Samples prepared via Loopamp^TM^ PURE DNA extraction kit (Eiken Chemical Co.).*

### Evaluation Using Clinical Strains

Using the 14 clinical *P. aeruginosa* strains, the GES-LAMP assay amplified the five *bla*_GES_ segments (GES-1, -5, -7, -9, and -19/20-like; Table 2), and no other genotype. The results were thus identical to those of PCR (Table 2 and Supplementary Figure S3).

### Carba-GES-LAMP Assay

Of the five *bla*_GES_-positive samples, only GES-5 and -19/20-like β-lactamase-producing isolates exhibited carbapenem resistance (Table 2), consistent with their genotypes. Our Carba-GES-LAMP assay detected only GES-5 and -19/20-like alleles containing the *bla*_GES_ Gly165Ser missense mutation. Using the ARMS, we successfully designed the Carba-GES-LAMP primers that distinguish the GES variants. The detection limit of the Carba-GES-LAMP assay was 10^4^ DNA copies. No amplification of non-carbapenemase-producing *bla*_GES_ genotypes (GES-1, -7, and -9) or any other genotype was evident within 50 min. The product was subjected to direct sequencing. The sequences were compared with those of the targeted region (bases 450–512) of the GES-5 gene (from F1 to B1c, Supplementary Figure S1B). The five base pairs of 3′ end of B1c region were matching to the Carba-GES-LAMP primer sequences (ACCGT; 493-497, Supplementary Figure S1B) including two mutations (Table 3) and obtained other sequences were identical to those expected (Supplementary Figure S2B).

## Discussion

We established a novel LAMP assay detecting *bla*_GES_. Because β-lactamase genes spread widely via plasmids, rapid and accurate POCT drug-resistance assessment is imperative. Our *bla*_GES_ LAMP assay was highly specific and more sensitive than PCR. *bla*_GES_ was correctly identified in 14 clinical isolates expressing various β-lactamases. LAMP reactions are not inhibited by contaminants in DNA-spiked samples. On the other hand, PCR reactions are inhibited by such contaminants, especially heparin (Satsangi et al., 1994), and other blood components including heme, leukocyte DNA, and immunoglobulin G; such inhibitors must be removed prior to PCR (Al-Soud et al., 2000; Al-Soud and Radstrom, 2001). We used the Loopamp^TM^ PURE DNA extraction kit (Eiken Chemical Co.) to extract DNA over 30 min, and centrifugation was not required. LAMP can thus be performed at the bedside. We then sought to detect the G493A mutation. GES β-lactamases hydrolyse oxyimino-cephalosporins and those with the Gly165Ser mutation exhibit carbapenemase activity (Naas et al., 2016). *P. aeruginosa bla*_GES_ strains may cause fatal nosocomial diseases. An outbreak of GES-5 β-lactamase-producing *P. aeruginosa* was reported in a long-term Japanese care facility (Kanayama et al., 2016). We designed GES-type carbapenemase-specific primers via ARMS, first demonstrated in 1989 (Newton et al., 1989) to detect any mutation via PCR. In 2007, Ikeda et al. used ARMS to design LAMP primers and detected a point mutation predicting the effects of the anti-lung cancer drug gefitinib (Ikeda et al., 2007). We previously used ARMS to establish a LAMP assay that distinguished *Neisseria meningitidis* serogroup Y from serogroup W (Lee et al., 2015). Here, we used ARMS to design the BIP, avoiding amplification of the wild-type gene. LAMP may be useful to detect GES-type carbapenemase genes.

## Conclusion

We established a novel LAMP assay for *bla*_GES_ with a comparable specificity and greater sensitivity to those of PCR. LAMP assays do not require substrate purification and are appropriate for POCT. LAMP can be used to detect GES-type carbapenemase genes with missense mutations. Further work involving more clinical specimens is required.

## Author Contributions

CT, MS, DK, HG, RM, KK, and PK contributed the conception of this study. CT and MS designed the experiments. HG, RM, KK, and PK acquired the samples. CT and MS analyzed the data. CT, MS, DK, and SH interpreted the data, drafted the manuscript, and approved the manuscript.

## Conflict of Interest Statement

HG is an employee of Evelo Biosciences. The remaining authors declare that the research was conducted in the absence of any commercial or financial relationships that could be construed as a potential conflict of interest.
