# Supplementary material for: Development of a Novel Loop-Mediated Isothermal Amplification Method to Detect Guiana Extended-Spectrum (GES) β-Lactamase Genes in Pseudomonas aeruginosa
Source: Front Microbiol. 2019 Feb 4;10:25. doi: 10.3389/fmicb.2019.00025 (PMC6369207; doi:10.3389/fmicb.2019.00025)
Supplement: Supplementary file 1 [file Image_1.pdf]

Figure S1. Nucleotide sequence of the *bla*$_{GES}$ used to design the GES-LAMP (A) and Carba-GES-LAMP (B) primer sets. The sequences used for the LAMP primers are indicated by arrows. (A) and (B) in the figure are the sequences of *Pseudomonas aeruginosa* β-lactamase GES-1 (GenBank accession number, AF355189.1) and GES-5 (GenBank accession number, EF190326.1) genes, respectively.

(A)

```
No         211                                                                      280
Sequence   GCCGCGCTGG TCTTTGAAAG AATTGACTCA GGCACCGAGC GGGGGGATCG AAAACTTTCA TATGGGCCGG


No         281                                                                      350
Sequence   ACATGATCGT CGAATGGTCT CCTGCCACGG AGCGGTTTCT AGCATCGGGA CACATGACGG TTCTCGAGGC
Primer        |---------F3--------->              |---------F2---------> <-------LF---

No         351                                                                      420
Sequence   AGCGCAAGCT GCGGTGCAGC TTAGCGACAA TGGGGCTACT AACCTCTTAC TGAGAGAAAT TGGCGGACCT
Primer     ---|    <---------F1---------|                              |-------B1------

No         421                                                                      490
Sequence   GCTGCAATGA CGCAGTATTT TCGTAAAATT GGCGACTCTG TGAGTCGGCT AGACCGGAAA GAGCCGGAGA
Primer     ---->      |---------LB------------>     <---------B2-------|          <----

No         491                                                                      560
Sequence   TGGGCGACAA CACACCTGGC GACCTCAGAG ATACAACTAC GCCTATTGCT ATGGCACGTA CTGTGGCTAA
Primer     ----B3-------|

No         561                                                                      630
Sequence   AGTCCTCTAT GGCGGCGCAC TGACGTCCAC CTCGACCCAC ACCATTGAGA GGTGGCTGAT CGGAAACCAA
```

(B)

```
No         211                                                                      280
Sequence   GCCGCGCTGG TCTTTGAAAG AATTGACTCA GGCACCGAGC GGGGGGATCG AAAACTTTCA TATGGGCCGG

No         281                                                                      350
Sequence   ACATGATCGT CGAATGGTCT CCTGCCACGG AGCGGTTTCT AGCATCGGGA CACATGACGG TTCTCGAGGC

No         351                                                                      420
Sequence   AGCGCAAGCT GCGGTGCAGC TTAGCGACAA TGGGGCTACT AACCTCTTAC TGAGAGAAAT TGGCGGACCT
Primer                    |---------F3-------->                      |-------F2---------

No         421                                                                      490
Sequence   GCTGCAATGA CGCAGTATTT TCGTAAAATT GGCGACTCTG TGAGTCGGCT AGACCGGAAA GAGCCGGAGA
Primer     ><----------LF----------|        <--------F1---------|

No         491                                                                      560
Sequence   TGAGCGACAA CACACCTGGC GACCTCAGAG ATACAACTAC GCCTATTGCT ATGGCACGTA CTGTGGCTAA
Primer       |---------B1--------->|-----------LB---------->< ---------B2---------|<----

No         561                                                                      630
Sequence   AGTCCTCTAT GGCGGCGCAC TGACGTCCAC CTCGACCCAC ACCATTGAGA GGTGGCTGAT CGGAAACCAA
Primer     <--------B3-------|
```
