# Supplementary figures and images for: Development of a Novel Loop-Mediated Isothermal Amplification Method to Detect Guiana Extended-Spectrum (GES) β-Lactamase Genes in Pseudomonas aeruginosa
Source: Front Microbiol. 2019 Feb 4;10:25. doi: 10.3389/fmicb.2019.00025 (PMC6369207; doi:10.3389/fmicb.2019.00025)

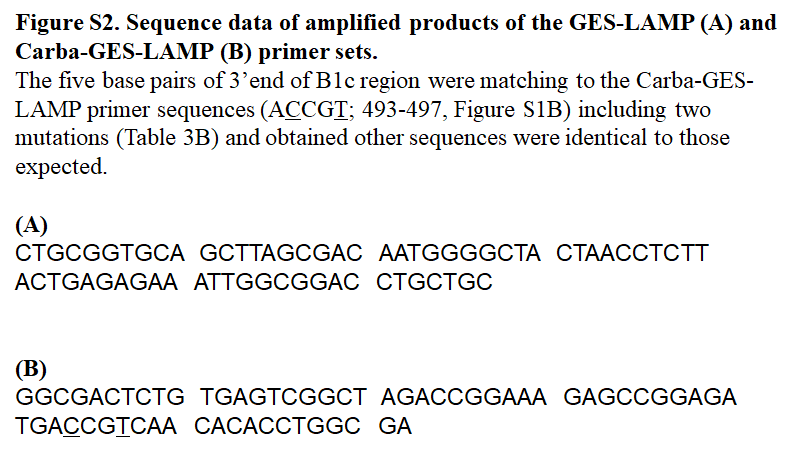

Supplement: Supplementary file 2 [file Image_2.tif]

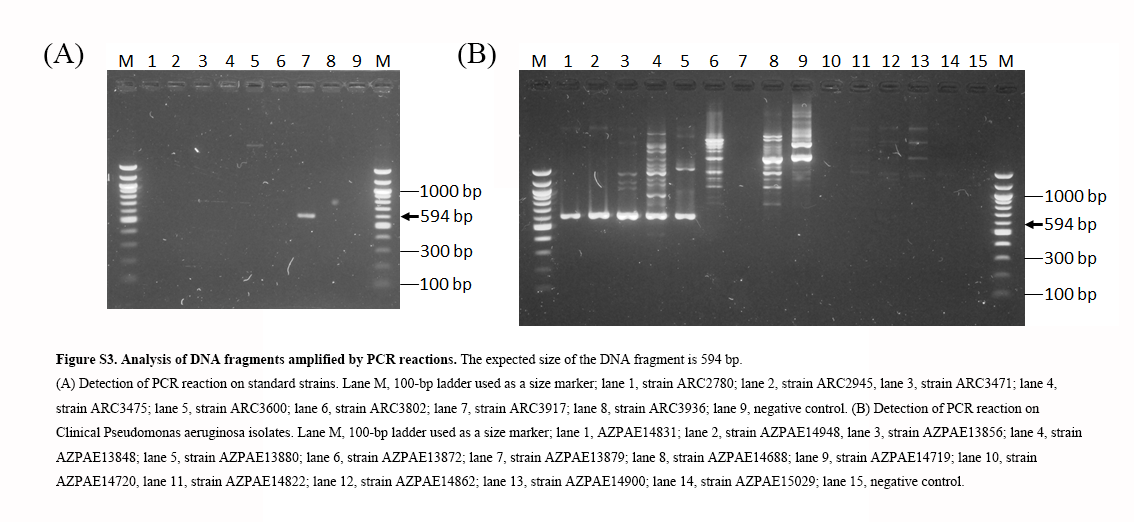

Supplement: Supplementary file 3 [file Image_3.tif]
